# Neuroendocrine tumors of colon and rectum: validation of clinical and prognostic values of the World Health Organization 2010 grading classifications and European Neuroendocrine Tumor Society staging systems

**DOI:** 10.18632/oncotarget.13641

**Published:** 2016-11-26

**Authors:** Chaoyong Shen, Yuan Yin, Huijiao Chen, Sumin Tang, Xiaonan Yin, Zongguang Zhou, Bo Zhang, Zhixin Chen

**Affiliations:** ^1^ Department of Gastrointestinal Surgery, West China Hospital, Sichuan University, Chengdu 610041, Sichuan, China; ^2^ Department of Pathology, West China Hospital, Sichuan University, Chengdu 610041, Sichuan, China; ^3^ Institute of Digestive Surgery and State Key Laboratory of Biotherapy, West China Hospital, Sichuan University, Chengdu 610041, Sichuan, China

**Keywords:** neuroendocrine tumors, colorectal, prognosis, WHO classifications, ENETS TNM systems

## Abstract

**Background/Aims:**

This study evaluated and compared the clinical and prognostic values of the grading criteria used by the World Health Organization (WHO) and the European Neuroendocrine Tumors Society (ENETS). Moreover, this work assessed the current best prognostic model for colorectal neuroendocrine tumors (CRNETs).

**Results:**

The 2010 WHO classifications and the ENETS systems can both stratify the patients into prognostic groups, although the 2010 WHO criteria is more applicable to CRNET patients. Along with tumor location, the 2010 WHO criteria are important independent prognostic parameters for CRNETs in both univariate and multivariate analyses through Cox regression (*P*<0.05).

**Methods:**

Data from 192 consecutive patients histopathologically diagnosed with CRNETs and had undergone surgical resection from January 2009 to May 2016 in a single center were retrospectively analyzed.

**Conclusions:**

Findings suggest that the WHO classifications are superior over the ENETS classification system in predicting the prognosis of CRNETs. Additionally, the WHO classifications can be widely used in clinical practice.

## INTRODUCTION

Colorectal neuroendocrine tumors (CRNETs) are a group of heterogeneous neoplasms traditionally referring to carcinoid tumors [1–3]. The annual incidence of rectal neuroendocrine tumors (NET) is approximately 0.86/100,000. Despite the obviously increasing incidence of CRNETs in recent decades, these tumors remain uncommon, accounting for ∼20% of all NETs [4–6]. Moreover, the overall incidence of CRNETs is slightly higher in males than in females [7].

CRNETs are heterogeneous and rare, and their stratification into different prognostic groups has been hindered by the lack of a unified staging system; NET has been evolving for more than a century already [8–9]. In 2000, the World Health Organization (WHO) established the first classification system, which is based on clinicopathological characteristics of NET. In 2010, this classification was updated and redefined into four categories: NET G1, NET G2, neuroendocrine carcinoma G3 (NEC G3), and mixed adenoneuroendocrine carcinomas (MANEC) [10]. As for the prediction of short-term prognosis, the WHO 2010 grading was found to be superior to the WHO 2000 grading [11]. Moreover, the 2010 WHO 2010 grading system is a prognostic factor for the survival of NET as validated by several studies [12–14]. The European Neuroendocrine Tumor Society (ENETS) proposed a tumor-node-metastasis (TNM) staging system in 2006 [15–16]. The prognostic significance and the stage-specific survivals according to ENETS TNM staging systems have been confirmed by some studies [17–19].

The 2010 WHO grading classifications differ from the ENETS TNM staging systems. The former define the diverse biology of NETs, reflecting the tumor's inherent malignant potential to a certain extent. By contrast, the latter merely reflected a time point in the disease process. Given the different emphases of these two schemes, concerns on the potential confusion may be raised in clinical NET management. A new staging system that encompasses all of the elements of these two classifications is undeniably urgently needed.

The ability of the 2010 WHO grading classifications and the ENETS classification systems to predict the prognosis of CRNETs after surgery has not yet been explored. Whether any of these systems is optimal for clinical use remains unknown. Therefore, on the basis of the data obtained from 192 consecutive patients in our institution, we attempted to clarify the clinicopathologic features of CRNETs to evaluate the clinical consistency of the 2010 WHO grading classifications and the ENETS TNM systems. Additionally, we explored the prognostic power of both systems for survival analyses of CRNETs.

## RESULTS

### Patient characteristics

A total of 192 eligible and consecutive CRNETs patients were identified in this cohort study, and their clinicopathologic features are summarized in Table 1. Of the 192 patients, 108 (56.25%) were males and 84 (43.75%) were females, and the male-to-female ratio is 1.29:1. The median age at initial diagnosis was 60 years (range: 24–88 years old), and the mean age is 57.71±13.55 years. The average size of the primary CRNETs was 1.45±1.63 cm with a median of 0.80 cm (range: 0.10–8.50 cm). Of these patients, 137 (71.35%) were incidentally found through screening colonoscopy and then based on abdominal discomfort/pain and change in bowel habits. Radical resection (R0) was performed in 184 patients, whereas 8 patients underwent R1/R2 resection. In terms of the ENETS TNM staging systems, stages I, II, III, and IV were detected in 136 (70.83%), 17 (8.85%), 32 (16.67%), and 7 (3.65%) patients, respectively. A total of 132 (68.75%) tumors were classified as NET G1, 15 (7.81%) as NET G2, 36 (18.75%) as NEC G3, and 9 (4.69%) as MANEC according to the 2010 WHO grading schemes.

**Table 1:** Clinicopathological features of surgically resected colorectal neuroendocrine tumors in our cohort research (n=192)

Variables	Mean±SD (Number/Percentage)
Gender	
Male	108 (56.25)
Female	84 (43.75)
Age (years)	57.71±13.55
Median (range)	60 (24∼88)
Tumor size (cm)	1.45±1.63
Median (range)	0.80 (0.10∼8.50)
Tumor location	
Colon	14 (7.29)
Rectum	178 (92.71)
Hospital stay (days)	8.51±7.49
Surgical approach	
Open surgery	53 (27.60)
Endoscopic/transanal resection	139 (72.40)
Surgical margin	
R0	184 (95.83)
R1/R2	8 (4.17)
Co-morbidity (Yes/No)	19 (9.90)/173 (90.10)
Vascular invasion (Yes/No)	9 (4.69)/183 (95.31)
TNM staging by ENETS criteria	
I	136 (70.83)
II	17 (8.85)
III	32 (16.67)
IV	7 (3.65)
WHO 2010 grading	
NET G1	132 (68.75)
NET G2	15 (7.81)
NEC G3	36 (18.75)
MANEC	9 (4.69)

SD: standard deviation; co-morbidity: including synchronous with other malignant tumor, diabetes mellitus, severe chronic cardiopulmonary disease, cerebrovascular disease, and chronic renal dysfunction. NET G1: neuroendocrine tumors G1; neuroendocrine tumors G2: NET G2; neuroendocrine carcinoma G3: NEC G3; mixed adenoneuroendocrine carcinomas: MANEC.

### Analysis using the 2010 WHO grading system and tumor location

With a median follow-up time of 32 months (mean: 37.43±25.37 months; range: 2–90 months), 27 patients showed tumor recurrence/distant metastasis. Of these 27 patients, 0, 2, 19, and 6 cases were NET G1, NET G2, NEC G3, and MANEC, respectively. As for the clinicopathologic differences among all grading groups, no significant difference in mean age was observed at initial diagnosis (*P*=0.585, Table 2). The number of male patients with MANEC is higher than the number of female patients; the opposite trend was observed in NET G1/G2 and NEC G3 (*P*=0.030) patients. NET G1/G2 were smaller than NEC G3 and MANEC, respectively (*P*<0.001). NET G1/G2 and MANEC were frequently observed in the rectum, whereas NEC G3 was frequently observed in the colon (*P*<0.001). Moreover, most NET G1/G2 cases were observed in stage I/II patients (130/132 and 12/15, respectively), whereas NEC G3 and MANEC cases were mainly observed in stage III/IV patients (27/36 and 7/9, respectively, *P*<0.001). Lymph node metastasis was found in 34 (15.6%) patients in this cohort (NET G1 in 1 patient, NET G2 in 3 patients, NEC G3 in 23 patients, and MANEC in 7 patients). All patients underwent surgical treatment and were postoperatively diagnosed with NETs arising from the colon (n=14) or rectum (n=178). Colonic NETs were significantly larger than rectal NETs (*P*<0.001). Moreover, distribution of T/N and TNM stages differed between colonic and rectal NETs. Most of the colonic NETs were classified as T3/T4, N1 tumor, and stage III/IV (*P*≤0.001). However, no significant difference was observed between colonic and rectal NETs at M stage (*P*=0.084). The overall mean hospital stay of colonic and rectal NET patients was 12.79±5.47 and 8.17±7.54 days, respectively, and the difference was notable (*P*=0.026, Table 3).

**Table 2:** Distributions of colorectal neuroendocrine tumors with different grades based on WHO 2010 Classifications

Variables	NET G1 (n=132, %)	NET G2 (n=15, %)	NEC G3 (n=36, %)	MANEC (n=9, %)	*P* value
Gender					0.030
Male	75 (56.82)	10 (66.67)	23 (63.89)	1 (11.11)	
Female	57 (43.118)	5 (33.33)	13 (36.11)	8 (88.89)	
Age (years)	58.45±13.68	58.60±13.17	55.28±13.77	55.11±11.87	0.585
Tumor size (cm)	0.72±0.59	1.45±1.15	3.45±1.98	4.06±1.94	<0.001
Tumor location					<0.001
Colon	1 (0.76)	2 (13.33)	11 (30.56)	0 (0.00)	
Rectum	131 (88.24)	13 (86.67)	25 (69.44)	9 (100.00)	
Surgical margin					0.001
R0	131 (88.24)	15 (100.00)	31 (86.11)	7 (77.78)	
R1/R2	1 (0.76)	0 (0.00)	5 (13.89)	2 (22.22)	
Vascular invasion	1 (0.76)	3 (20.00)	4 (11.11)	1 (11.11)	0.001
TNM stages					<0.001
I	119 (90.15)	9 (60.00)	6 (16.67)	2 (22.22)	
II	11 (8.33)	3 (20.00)	3 (8.33)	0 (0.00)	
III	1 (0.76)	3 (20.00)	22 (61.11)	6 (66.67)	
IV	1 (0.76)	0 (0.00)	5 (13.89)	1 (11.11)	

NET G1: neuroendocrine tumors G1; neuroendocrine tumors G2: NET G2; neuroendocrine carcinoma G3: NEC G3; mixed adenoneuroendocrine carcinomas: MANEC; TNM: tumor-node-metastasis.

**Table 3:** Comparison of clinicopathological characteristics between colonic and rectal neuroendocrine tumors

Variables	Colonic NET (n=14, %)	Rectal NET (n=178, %)	*P* value
Gender (M/F, cases)	8/6	100/78	0.944
Age (years)	55.50±14.77	57.89±13.48	0.527
Tumor size (cm)	4.41±2.04	1.21±1.34	<0.001
Hospital stay (days)	12.79±5.47	8.17±7.54	0.026
Vascular invasion	2 (14.29)	7 (3.93)	0.132
Surgical margin			0.461
R0	13 (92.86)	171 (96.07)	
R1/R2	1 (7.14)	7 (3.93)	
T stage			<0.001
T1	1 (7.14)	141 (79.21)	
T2	1 (7.14)	11 (6.18)	
T3	7 (50.00)	11 (6.18)	
T4	5 (35.71)	15 (8.43)	
N stage			0.001
N0	6 (42.86)	152 (85.39)	
N1	8 (57.14)	26 (14.61)	
M stage			0.084
M0	12 (85.71)	173 (97.19)	
M1	2 (14.29)	5 (2.81)	
TNM staging by ENETS criteria			<0.001
I	0 (0.00)	136 (76.40)	
II	4 (28.57)	13 (7.30)	
III	8 (57.14)	24 (13.48)	
IV	2 (14.28)	5 (2.81)	

NET: neuroendocrine tumors; M: male; F: female; TNM: tumor-node-metastasis; ENETS: the European Neuroendocrine Tumors Society.

### Survival rates according to the ENETS and the 2010 WHO criteria

**Figure 1:**
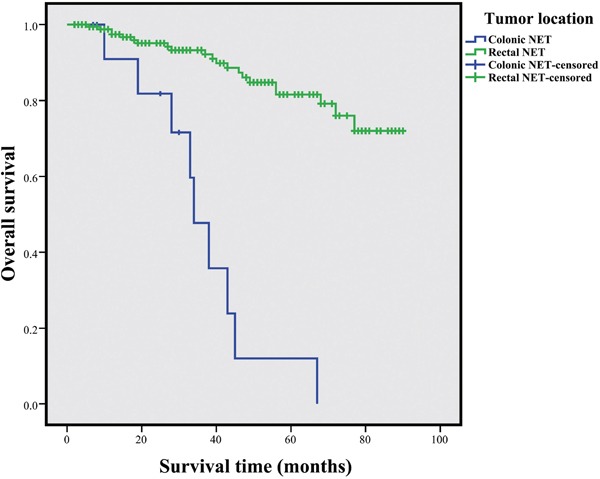
Comparison of survivals for CRNETs stratified by tumor location The colonic NET exhibited a worse prognosis than that of rectal NETs (3-year of OS: 47.7% vs 93.3%, respectively; 5-year of OS: 11.9% vs 81.6, respectively, *P*<0.001).

**Figure 2:**
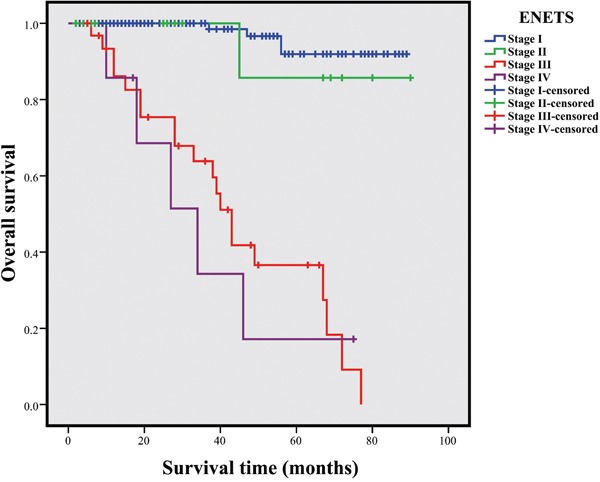
Survivals for CRNETs in different stages by the ENETS staging systems Difference of survival among four groups was statistical significance (*P*<0.001).

By the end of the follow-up period (June 2016 to July 2016), 30 patients died because of NET progression or any other reasons. The 1-, 3-, and 5-year OS rates for the entire cohort were 97.0%, 89.7%, and 75.6%, respectively. The colonic NETs exhibited a worse prognosis than the rectal NETs (3-year OS: 47.7% vs. 93.3%, respectively; 5-year OS: 11.9% vs. 81.6, respectively, *P*<0.001, Figure 1). A TNM stage was assigned for each patients according to the ENETS staging systems. Based on this criteria, 142, 12, 18, and 20 tumors were defined as T1, T2, T3, and T4, respectively. Lymph node metastasis occurred in 34 patients, and 7 patients showed distant metastasis. As for the ENETS system, the 5-year OS rates in stages I, II, III, and IV were 91.9%, 85.7%, 36.6%, and 17.1%, respectively (Figure 2). Significant differences in survival were observed among stages I, III, and IV patients (*P*<0.001 and *P*<0.001, respectively) and among stages II, III, and IV patients (*P*=0.001 and *P*=0.004, respectively). However, the OS of stage I and II patients did not significantly differ (*P*=0.539), whereas that of stages III and IV patients significantly differed (*P*=0.635).

**Figure 3:**
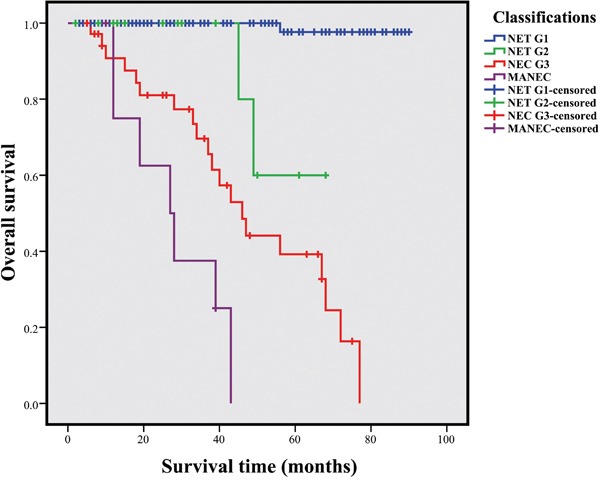
Survivals of CRNETs with different grades according to the WHO 2010 grading classifications (P<0.001)

Based on the 2010 WHO criteria, the 5-year OS rates of NET G1, NET G2, NEC G3, and MANEC were 97.7%, 60.0%, 39.2%, and 0.0%, respectively (Figure 3). OS of NET was higher than that of NET G2, NEC G3, and MANEC (*P*<0.001). In addition, NEC G3 showed a better OS than MANEC (*P*=0.023); OS was longer in NET G2 than in MANEC (*P*<0.001), and survival time between NET G2 and NEC G3 did not significantly vary (*P*=0.102). The mean OS time for NET G1, NET G2, NEC G3, and MANEC groups were 39.26±27.04, 31.67±19.93, 36.14±22.64, and 25.44±12.88 months, respectively.

### Prognostic factors associated with OS

**Figure 4:**
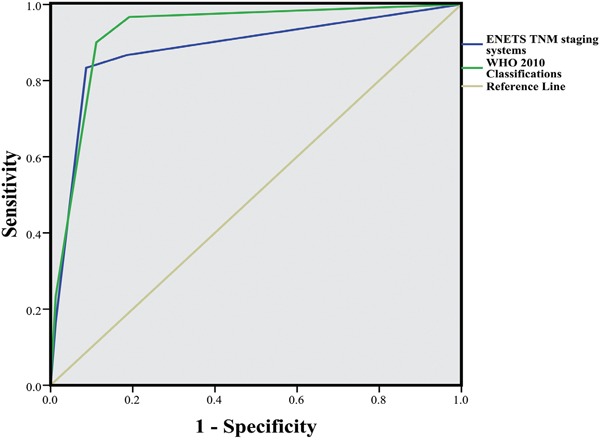
ROC curves compare the prognostic accuracy between the WHO 2010 classifications and ENETS TNM systems The area under the curve were respective 0.927 (95% CI: 0.879-0.976) and 0.883 (95% CI: 0.804-0.961) for the WHO 2010 criteria and ENETS TNM systems.

The potential clinicopathological parameters affecting survivals of CRNETs were analyzed in this study. Univariate analysis of prognostic factors through the Kaplan–Meier method showed that OS was statistically associated with tumor size (≤0.8 cm vs. >0.8 cm, *P*<0.001), tumor location (colon vs. rectum, *P*<0.001), surgical margin (R0 vs. R1/R2, *P*<0.001), T stage (T1/T2 vs. T3/T4, *P*<0.001), N stage (N0 vs. N1, *P*<0.001), M stage (M0 vs. M1, *P*<0.001), vascular invasion (Yes vs. No, *P*=0.033), TNM stage (I/II vs. III/IV, *P*<0.001), and WHO grades (NET G1/G2 vs. NEC G3/MANEC, *P*<0.001). A cut-off value of 0.8 cm for tumor size was determined from the median value. By incorporating these factors, except the highly collinear variables, into the Cox multivariate regression proportional hazards model, we found that tumor location and the 2010 WHO classifications are independent predictors for CRNETs (Table 4). Moreover, an ROC curve was produced (Figure 4) to evaluate the performance of the 2010 WHO criteria and ENETS systems in terms of OS prediction. The ROC analysis showed that the area under the curve was 0.927 (95% CI: 0.879–0.976) for the 2010 WHO criteria, whereas 0.883 (95% CI: 0.804–0.961) for the ENETS TNM systems.

**Table 4:** Multivariate analysis of potential predictors associated with overall survival for surgically resected colorectal neuroendocrine tumors

Variables	Hazard ratio	95% confidence interval	*P* value
Tumor size			0.521
≤0.8 cm	Reference		
>0.8 cm	1.708	0.333-8.756	
Tumor location			0.003
Rectum	Reference		
Colon	4.672	1.714-12.733	
Surgical margin			0.737
R0	Reference		
R1/R2	1.257	0.330-4.791	
Vascular invasion			0.625
No	Reference		
Yes	1.342	0.413-4.354	
TNM stage			
I	Reference		
II	0.725	0.064-8.214	0.795
III	1.670	0.373-7.469	0.502
IV	2.232	0.39712.546	0.362
2010 WHO grading			
NET G1	Reference		
NET G2	15.477	2.179-42.623	0.011
NEC G3	32.262	5.513-70.314	0.001
MANEC	91.797	23.149-131.165	<0.001

TNM: tumor-node-metastasis; WHO: World Health Organization; NET G1: neuroendocrine tumors G1; neuroendocrine tumors G2: NET G2; neuroendocrine carcinoma G3: NEC G3; mixed adenoneuroendocrine carcinomas: MANEC.

## DISCUSSION

NETs, which originate from various tissues, are tumors showing a wide spectrum of biological behaviors ranging from benign to malignant [1, 4, 7]. Given their rarity and heterogeneity, however, NETs have not yet been extensively studied. The increasing incidence and diagnosis of NET has highlighted the need for a uniform staging system for disease prognosis. Studies have investigated the prognostic significance of gastroenteric/pancreatic NET [13, 14, 19]; by contrast, colorectal NETs are rarely investigated, especially in validating the prognostic power of the new WHO criteria and ENETS TNM classifications [12, 20]. The present study is the first to explore the clinical consistency of the 2010 WHO criteria and the ENETS systems on the survivals of CRNET patients who received surgical treatment. Additionally, this study validated the prognostic value of both schemes for CRNETs by using the data obtained from our cohort.

The lack of an accepted staging system limits the accurate prediction of the survival of NETs, although many attempts have been made to uniformly treat NETs. A uniform staging system for NET classifications and prognostications was inexistent until 2006. Tumor-, node-, and metastasis-based staging systems were first proposed by Rindi et al. [15] in 2006; this staging system classifies NETs into four groups, and such classification system was adopted by ENETS. A new TNM staging system, which is applicable to NETs in ileum, appendix, colon, and rectum, was subsequently proposed by Rindi et al. [21] in 2007. Although the ENETS system is not the best system [14], it can provide good prognostic stratification of the stage groups of pancreatic NET and CRNETs [19, 21, 22]. However, these proposals, which are meant to help clinicians in the stratification and management of NET patients, must be validated or evaluated. Moreover, the prognostic value of histological grading has been rigorously corroborated in previous studies. Thus, in 2000, WHO officially proposed a grading system and divided NETs into three main types. This classification was not widely accepted because of its low ability to predict the biological aggressiveness of NETs. Updating of this criterion is evidently necessary. A new grading scheme was proposed and accepted by the WHO in 2010 (i.e., the 2010 WHO grading classifications) [10]. Earlier works have validated the clinical and prognostic value of these new WHO classifications [12, 20, 23], although this new scheme has shown a low prognostic value for gastric NET [14]. To our knowledge, the clinical value and prognostic significance of CRNETs according to the updated WHO criteria and ENETS TNM systems have not yet been evaluated.

In 2015, Kim et al. [24] reported that among 175 patients with gastric NET diagnosed in a single institution, no mucosal or submucosal invasion or lymph node metastasis was observed in patients with G1 or G2 (≤1 cm) according to WHO classifications system. Thus, they advocated for the endoscopic resection or minimally invasive treatment of these lesions. By contrast, Kojima et al. [12] demonstrated that up to 26.5% of rectal NET metastasized in the lymph node, forming tumors of smaller than 20 mm. In addition, 9.2% of lymph node metastases were found in tumors smaller than 10 mm and that lymph node metastases were more frequent in NET G2 than in NET G1. Similar findings were reported by Konishi et al. [25], wherein 7% of lymph node metastases were observed in colorectal carcinoids smaller than 1 cm. Consistent with these results, the current findings indicated that NET G1 (0.72±0.59 cm) or NET G2 (1.45±1.15 cm) were small but still showed lymph node metastasis (1/132 and 3/15, respectively). Thus, the indications for minimally invasive surgery should be re-examined. This finding may be attributed to the fact that the potential biological behavior of NETs originating from different body parts may vary markedly even within the same WHO grade. Moreover, site-dependent histological variations were found; as reported, 91.4%, 30.0%, and 25.0% of rectal, colonic, and appendiceal NETs, respectively, were NET G1 [12]. By contrast, our result showed a relatively low proportion of NET G1 in rectal or colonic NETs (131/178 vs. 1/14, respectively), and NET G1 was predominant in rectal NETs. In addition, our study revealed that NET G2 showed frequent vascular invasion, whereas Kojima et al. [12] reported that NET G2 demonstrated frequent lymph node metastasis.

The stage-specific 5-year survival of CRNETs was previously determined. Yao et al. [4] reported that the 5-year survival of localized, regionally, and distantly metastatic colonic NETs were 85%, 46%, and 14%, respectively, whereas 90%, 62%, and 24% for rectal NET, respectively. In 2013, Weinstock et al. [20] evaluated the clinical and prognostic characteristics of rectal NET, and they concluded that the median OS of 141 patients was 6.8 years (range: 0.8–34.7 years), with a 5-year OS rate of 84.4%, similar to our findings. Moreover, Chagpar et al. [26] showed that the ENETS system can discriminate the outcomes of CRNETs patients, and the 5-year OS rates of stages I, II, III, and IV based on this classification were 90.8%, 77.3%, 53.1%, and 14.8%, respectively. Survival analysis stratified by tumor location was also performed, and the 5-year OS of colonic and rectal NET in stages I, II, III, and IV were 86.6% versus 91.7%, 69.4% versus 91.7%, 54.0% versus 44.9%, and 15.3% versus 11.8%, respectively. Consistent with their findings [20, 26], the present result demonstrated that CRNETs patients could be stratified by the ENETS systems into different prognostic groups (*P*<0.001, Figure 2). However, some survival curves intertwined with each other. Stage I patients showed a better survival than the stages III and IV patients (*P*<0.001 and *P*<0.001, respectively), as well as those between stage II and III or IV (*P*=0.001 and *P*=0.004, respectively). Moreover, the survival of stages I/II patients (*P*=0.539) and stages III/IV patients (*P*=0.635) did not significantly differ. Therefore, to a certain extent, the OS of CRNETs was not well distributed based on ENETS TNM staging systems.

The new 2010 WHO classifications were previously validated [11, 12, 20, 27, 28]; in this classification, patients were classified into four groups with different survival rates. The 5-year OS in patients with rectal NET G1–G3 tumors were 87.7%, 47.6%, and 33.3%, respectively [20]. In 2014, Lee et al. [11] reported that the 5-year of OS of 34 CRNETs patients were 78%±14% in G1, 40%±30% in G2, and 31%±12% in G3 (G1 vs. G3, *P*=0.028; G1 vs. G2, *P*=0.41; and G2 vs. G3, *P*=0.37). Similarly, the grade-stratified 5-year OS rates of NET G1, NET G2, and NEC G3 derived from our center were 97.7%, 60.0%, and 39.2%, respectively. NET G1 showed a significantly longer survival time than NET G2, NEC G3, and MANEC (*P*<0.001), whereas the survival time between NET G2 and NEC G3 did not significantly vary (*P*=0.102).

Many factors, such as primary tumor location, size, extent of surgical resection, TNM stages, histologic grade, lymphovascular invasion, extent of disease, and liver metastasis, are associated with prediction of CRNET prognosis [11, 25, 26, 28–30]. Kim et al. [28] demonstrated that colonic NETs in patients are negative predictors of CRNETs. Moreover, Chagpar et al. [26] reported that T, N, and M stages, histologic grade, and tumor size, and race and year of diagnosis, exert a significant prognostic effect. In the present study, univariate analysis revealed that tumor size, tumor location, surgical margin, T, N, and M stages, vascular invasion, TNM stage, and WHO grades were statistically associated with OS of CRNETs (*P*<0.05), whereas gender, age at initial diagnosis, and co-morbidity did not significant affect the OS. In multivariate analysis, only tumor location and the 2010 WHO classifications were the independent prognostic factors for OS of CRNETs. This result was observed probably because factors such as tumor size and TNM stages may somehow affect OS of CRNETs, although their role as independent prognostic indicators is not as significant as that of the other factors. Thus, our study preliminarily demonstrated the clinical and prognostic values of the 2010 WHO classifications.

However, our study had several limitations. First, the major limitation of this research is its retrospective nature with inherent bias. In addition, the sample size for NET G2 and MANC is small, although a convincing conclusion was still drawn from such a small sample. A multicenter, large-scale trail is thus needed to further validate the prognostic value of this scheme. The 2010 AJCC staging systems have also been used in clinics and have been demonstrated as an independent predictor for survival analysis of CRNETs [26]. However, the present study investigated the clinical and prognostic value of the 2010 WHO 2010 criteria and ENETS systems only. Last but not the least, the 2010 WHO classifications cannot completely evaluate the biological behavior of tumors, and future modification of these proposed staging systems are warranted. A more applicable staging system, such as combined WHO criteria and TNM staging systems used in devising a new tumor-grading-metastasis staging system [31] or in finding novel markers of diseases, is also needed.

In summary, we analyzed the clinical and prognostic values of CRNETs by using the 2010 WHO criteria and ENETS staging systems. Our results suggested that the WHO classifications are superior over the ENETS systems in predicting the prognosis of CRNETs; moreover, the results supported the wide use of these systems in clinical practice. Additionally, apart from the WHO criteria, tumor location also demonstrated a prognostic value for CRNETs. However, this factor is not currently incorporated into any staging schemes, and in-depth studies are warranted.

## MATERIALS AND METHODS

### Patient selection

Data were obtained from the medical records of 192 consecutive patients treated in West China Hospital of Sichuan University from January 2009 to May 2016. All patients were surgically treated (endoscopic/transanal resection and open surgery) and their conditions were pathologically confirmed as CRNETs. Patients showing the clinical signs of CRNETs but were not pathologically diagnosed as such, as well as those showing tumor recurrence, were excluded in this study. The pathological specimens that cannot be classified according to the 2010 WHO criteria were also excluded. All neoplasms were sporadic and originated only from the colorectal region of the gut. The Institutional Review Board and Ethics Committee of the West China Hospital of Sichuan University deemed that an ethical review was not needed for this retrospective analysis. Written informed consent was obtained from all patients.

### Tumor characteristics and data collection

NET is defined based on the 2010 WHO classifications as follows: NET G1 (mitotic count is <2/10 under high-power fields and/or Ki-67 index is ≤2%), NET G2 (mitotic count is 2–20/10 under high-power fields and/or Ki-67 index is 3%–20%), NEC G3 (mitotic count is >20/10 under high-power fields and/or Ki-67 index is >20%), and MANEC. The MANEC contains neuroendocrine cells mixed with non-endocrine components (usually adenocarcinoma structures), and 30% of either component is required. All specimens were reexamined by a pathologist and reclassified based on the 2010 WHO criteria. Table 5 shows the definition of the ENETS TNM classification systems. Data include the patients’ demographics (age and gender), clinical symptoms, tumor size, primary tumor location, lymph invasion, distant metastasis, mitotic count, Ki-67 positive rate, surgical margin (R0: complete gross and microscopic resection; R1: microscopic residual lesions, and R2: presence of any gross residual tumors), co-morbidity (including those synchronous with other malignant tumors, diabetes mellitus, severe chronic cardiopulmonary disease, cerebrovascular disease, and chronic renal dysfunction), TNM stages at initial diagnosis, and survival outcomes.

**Table 5:** Proposed TNM staging system for endocrine tumors of colon and rectum (European Neuroendocrine Tumor Society)

T	Primary tumor
T x	Primary tumor cannot be assessed
T0	No Primary tumor
T1	Tumor invades mucosa or submucosa and size ≤2 cm
T2	Tumor invades muscularis propria or size >2 cm
T3	Tumor invades into subserosa, pericolic, and perirectal fat
T4	Tumor invades peritoneum or other organs
N	Regional lymph nodes
Nx	Regional lymph nodes cannot be assessed
N0	No regional lymph nodes metastases
N1	Regional lymph nodes metastases
M	Distant metastases
Mx	Distant metastases cannot be assessed
M0	No distant metastases
M1	Distant metastases
Stages	
I	T1N0M0
II	T2 or T3N0M0
III	T4N0M0 or TanyN1M0
IV	TanyNanyM1

### Follow-up and statistical analyses

Follow-up was conducted through clinic visit, telephone call, or outpatient clinic visit from June 2016 to July 2016. We lost contact to 23 patients and thus they were excluded from the final survival analysis. Overall survival (OS) is the time elapsed from the date of diagnosis to the date of death of any cause or to the date of last follow-up visit. Numerical data were expressed as mean ± standard deviation or median for quantitative variables analyzed using one-way ANOVA. Differences among groups were analyzed using ANOVA for continuous variables and χ^2^ test or Fisher's exact test for categorical data. Wilcoxon test was used to test ranked data. Survival data were analyzed using the Kaplan–Meier method along with log-rank test. Multivariate analyses using the Cox proportional hazards model were performed to assess the relative impact of clinicopathologic parameters on OS and to identify independent factors associated with prognosis. Significance was determined at 95% confidence interval (CI). Logistic regression combined with receiver operating characteristics (ROC) curve analysis was applied to investigate the prognostic accuracy of the two staging systems. A *P* value of <0.05 in two-sided test indicated significant difference. All statistical analyses were performed using Statistical Package for Social Science (SPSS Inc., Chicago, IL, USA).

## Abbreviations

WHO: World Health Organization
CRNETs: colorectal neuroendocrine tumors
ENETS: European Neuroendocrine Tumors Society
NET G1: neuroendocrine tumor G1
NET G2: neuroendocrine tumor G2
NEC G3: neuroendocrine carcinoma G3
MANEC: mixed adenoneuroendocrine carcinoma
TNM: tumor-node-metastasis
OS: overall survival
CI: confidence interval
ROC: receiver operating characteristics.
